# A comprehensive study on MDP effects: Microshear bond strength and fatigue resistance in 4YSZ ceramics

**DOI:** 10.1590/0103-644020246091

**Published:** 2024-12-16

**Authors:** Lucas Saldanha da Rosa, Luiza Freitas Brum Souza, Gratcheva Falcão Chiapinotto, Telma de Souza Pires, Amanda Maria de Oliveira Dal Piva, Cornelis Johannes Kleverlaan, Gabriel Kalil Rocha Pereira

**Affiliations:** 1 Post-Graduate Program in Oral Sciences (Prosthodontics Unit), Faculty of Dentistry, Federal University of Santa Maria (UFSM), Santa Maria, Rio Grande do Sul, Brazil.; 2 Department of Dental Materials Science, Academic Centre for Dentistry Amsterdam (ACTA), Universiteit van Amsterdam and Vrije Universiteit, Amsterdam, North Holland, the Netherlands

**Keywords:** adhesion, zirconia, MDP, aging, survival

## Abstract

The aim of this study is to assess the presence of MDP at various stages of the bonding procedure, enhance the adhesive and mechanical behavior of cemented zirconia ceramics. Fifty ceramic slices (15 × 15 × 2 mm) and 48 discs (Ø= 10 mm, 1 mm thickness) were prepared, sintered, air-abraded with aluminum oxide, and allocated considering: 1) microshear bond strength (µSBS) between ceramic slices and luting agent cylinders (height= 1 mm, Ø= 1.2 mm); 2) fatigue behavior, ceramic discs paired and bonded onto fiber-epoxy resin discs (Ø= 10 mm, 2.5 mm thickness), and then mechanically tested (cyclic loading, starting at 400N, with increments of 100N, until failure). Four experimental groups were defined: Universal Primer (MDP primer+non-MDP resin cement - RC), Universal Adhesive (MDP adhesive+non-MDP RC), Cement (no primer+MDP RC), and Primer+Cement (MDP primer + MDP RC). For both outcomes, half of the specimens were tested after 24 hours, and half after aging. Bond strength data was analyzed via two-way ANOVA and Tukey post-hoc tests, while fatigue data went through Kaplan-Meier and Mantel-Cox post hoc tests. Regarding µSBS, aging impaired adhesion only for the Primer+Cement group (p<0.001). Universal Primer and Universal Adhesive showed the highest bond strength (p<0.05). Despite that, fatigue data indicates no significant differences (p>0.05). In conclusion, systems with MDP-containing components associated with non-MDP resin cement demonstrated enhanced adhesive capability for zirconia restorations. Nevertheless, no differences in terms of mechanical reinforcement were observed.

## Resumo

O objetivo deste estudo é determinar se a presença de MDP nos vários estágios do procedimento de adesão melhora o comportamento adesivo e mecânico de cerâmicas à base de zircônia cimentadas. Cinquenta fatias cerâmicas (15 × 15 × 2 mm) e 48 discos (Ø= 10 mm, 1 mm espessura) foram preparados, sinterizados, jateados com óxido de alumínio e alocados considerando: 1) resistência de união ao microcisalhamento (μSBS) entre fatias cerâmicas e cilindros de agentes de cimentação (altura= 1 mm, Ø= 1.2 mm); 2) comportamento em fadiga, discos cerâmicos pareados e aderidos em discos de resina epóxi reforçados por fibra de vidro (Ø= 10 mm, 2.5 mm espessura), e então mecanicamente testados (fadiga cíclica, começando em 400N, com incrementos de 100N até a falha). Quatro grupos experimentais foram definidos: Primer Universal (primer com MDP + cimento resinoso - CR sem MDP), Adesivo universal (adesivo com MDP + CR sem MDP), Cimento (primer sem MDP + CR com MDP), e Primer+Cimento (primer com MDP + CR com MDP). Para ambos os desfechos, metade dos espécimes foram testados em 24 horas e metade após envelhecimento. Os dados foram submetidos aos seus respectivos testes estatísticos. Sobre μSBS, o envelhecimento prejudicou a resistência de união apenas no grupo Primer+Cimento (p<0.001). Primer Universal e Adesivo Universal apresentaram as maiores resistências de união (p<0.05). Apesar disso, os dados de fadiga não indicaram diferenças significativas (p>0.05). Em conclusão, sistemas contendo componentes com MDP associados a cimentos resinosos sem MDP demonstraram capacidade adesiva superior para restaurações de zircônia. Apesar disso, diferenças no reforço mecânico não foram observadas.

## Introduction

Over the years, zirconia ceramics have undergone modifications, evolving from being solely used as frameworks and copings to serving as monolithic restorations with enhanced aesthetics, particularly with 4 mol.% yttria-stabilized zirconia (4YSZ).^1^ However, due to the polycrystalline nature of zirconia, lacking a vitreous matrix and resistance to acid etching, establishing an effective adhesion protocol remains a challenge ^2^. The advantages of adhesive cementation, involving luting components with chemical adhesion to ceramic materials, are evident in improving the mechanical properties of glass ceramics, however, in a recent systematic review the same conclusion could not be drawn to zirconia ceramics ^3^.

Besides the zirconia’s great mechanical resistance ^4^, its polycrystalline nature makes it unable to bond with a traditional silane agent without a proper air abrasion with silica coated aluminum oxide ^5^. One concern about this technique is that the restoration surface may not be uniformly air abraded, leading to low-adhesive zones that could compromise the overall bond strength ^6^. Yet, the application of adhesive cementation in polycrystalline zirconia-based ceramics presents a knowledge gap that requires investigation ^3^. Notably, the impact of luting systems containing 10-Methacryloyloxydecyl dihydrogen phosphate (MDP) or similar substances in their components have been considered, given MDP's ability to form strong chemical interactions with metallic oxides, such as zirconium oxide ^7^. This occurs by bonding P-OH groups, present in MDP, to zirconia crystals. Consequently, it has been speculated that components with high MDP concentrations would enhance effectiveness in bonding to zirconia ^7^.

Recently, the market has seen the introduction of numerous luting systems incorporating MDP in various components. When opting for systems that feature this monomer, the clinicians have essentially three options, being one, systems with an MDP-containing primer or adhesive associated with an MDP-free cement. Another option is the use of an MDP-containing cement that does not require primers, and a third option with the use of an MDP-containing primer associated with an MDP-containing cement. Nevertheless, the optimal allocation of MDP within the luting components (primer, adhesive or cement) and whether the combination of different components containing it, yields additive effects when facing an environment of extensive challenges to the adhesive interface require further clarification ^8^. Moreover, when assessing the success of indirect restorations, crucial considerations include their adhesion and fracture resistance ^9^.

In the context of adhesion studies, employing longer and more aggressive water aging protocols is believed to yield more pertinent data for investigating bond interfaces ^10^. Aging in water-based media, besides breaking chemical bonds, can induce changes in the mechanical properties of cements ^8^. This alteration in properties influences stress distribution, potentially resulting in stress accumulation in ceramic restorations and leading to premature failures ^8^. Additionally, stress accumulation occurs in fatigue regimes, where subcritical loads are applied over extended periods. In such scenarios, minor defects can grow and become critical, diminishing the material's resistance and contributing to restoration failures ^11^.

Given the aforementioned, there is a lack of studies assessing luting strategies involving MDP at various steps with an intensive aging protocol concerning 4YSZ 's bond strength and mechanical fatigue behavior. Hence, this study investigated whether luting components featuring MDP at different stages of the cementation process and the combination of materials containing MDP could produce a favorable additive effect on microshear bond strength and fatigue behavior in simplified 4YSZ restorations bonded to an epoxy resin. The tested hypothesis were that luting systems containing MDP at the different components of the luting process, and the MDP additive effect on would improve microshear bond strength (first hypothesis) and fatigue behavior (second hypothesis) of simplified 4YSZ restorations bonded to an epoxy resin.

## Materials and Methods

**Figure 1.**
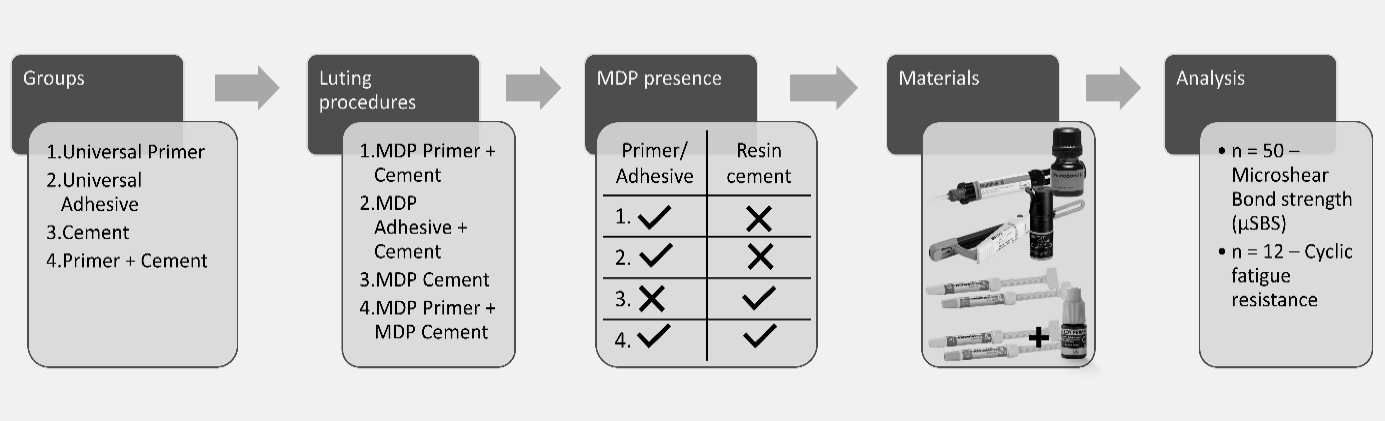
Study experimental design

The sample size was determined using G*Power (Heinrich-Heine-Universität, Düsseldorf, Germany), with parameters set at a power of 80%, an alpha-type error of 0.05, and an effect size of 0.42. The considered sample units for the microshear bond strength tests were the cement cylinders, while for the fatigue resistance tests were the luted assemblies. Details on the materials utilized, including manufacturers and composition information, are provided in Box 1. The study experimental design is outlined in Figure 1.

**Box 1:**
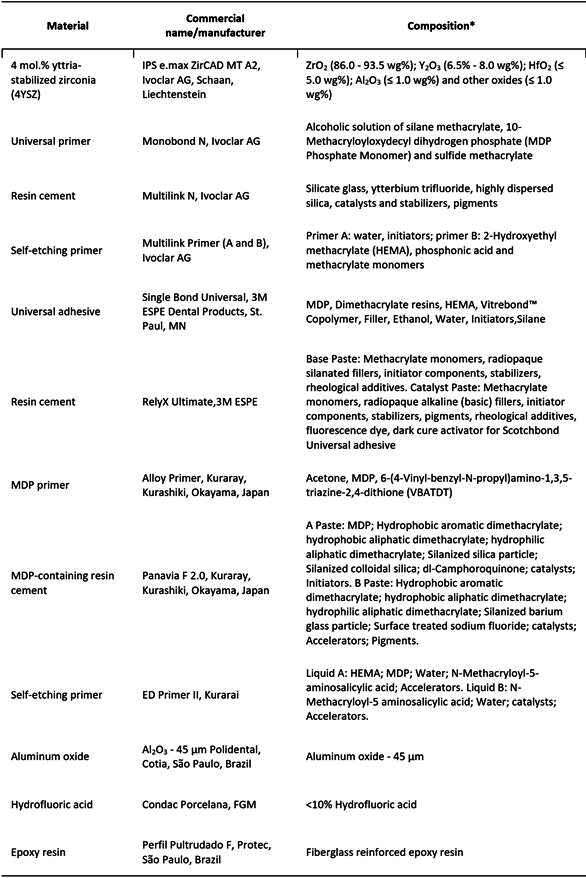
Details on the utilized materials, including commercial name, manufacturers, and composition information

*Data provided by the manufacturers

### Microshear bond stregth (µSBS) specimens

Forty zirconia (4YSZ) square slices, measuring 19 mm by 19 mm by 2.5 mm (width × height × thickness), were cut from prefabricated blocks (IPS e.max ZirCAD MT A2, Ivoclar, Schaan, Liechtenstein) using a cutting machine (IsoMet 1000, Buehler, Lake Bluff, USA) with continuous water-cooling. Topographical standardization was achieved by polishing with a sequence of silicon carbide sandpapers (#400-, #600- and #1200-grit) using a polishing machine (Ecomet/Automet 250, Buehler) under refrigeration for 30 s each sandpaper. Subsequently, the slices were sintered (VITA Zyrcomat 6000 MS, Vita Zahnfabrik, Germany) at 1,500°C for 120 min, following the manufacturer's recommendations, resulting in final dimensions of 15 mm by 15 mm by 2 mm (width × height × thickness). Finally, these slices were embedded in polyvinyl chloride (PVC) cylinders using self-curing acrylic resin, leaving the surfaces to be bonded exposed (JET Clássico, Campo Lindo Paulista, Brazil).

### Fatigue resistance specimens

Similar to the procedure for obtaining bond strength specimens, prefabricated 4YSZ blocks (IPS e.max ZirCAD MT A2, Ivoclar AG) were cut into squares measuring 13 mm by 13 mm using a cutting machine (IsoMet 1000, Buehler, Lake Bluff, USA) with water-cooling. These squares were then shaped into cylinders with a diameter of 12 mm using a polishing machine (Ecomet/Automet 250, Buehler). Subsequently, the cylinders were cut into 48 units of 1.2 mm thick discs using a cutting machine (IsoMet 1000, Buehler) under water-cooling, considering an estimated shrinkage rate of approximately 20% during the sintering process. Topographical standardization was achieved by polishing with silicon carbide sandpapers (#400-, #600- and #1200-grit) using a polishing machine (Ecomet/Automet 250, Buehler) under water-cooling for 30 s each sandpaper. The resulting discs were sintered following the same process as the slices, resulting in final dimensions of 10 mm in diameter and 1 mm thickness. Dentin analog discs (epoxy resin reinforced by glass fiber - Carbotec GmbH & Co. KG, Königs Wusterhausen, Germany), measuring 10 mm in diameter and 2.5 mm in thickness, were obtained through cutting large prefabricated cylinders through a similar process ^12^.

### Cleaning and surface treatments

Following preparation, the specimens underwent cleaning in an ultrasonic bath (92% isopropyl alcohol - 5 min). The epoxy resin discs were etched with 10% hydrofluoric acid for 60 s, followed by thorough washing with water and drying using an oil-free air jet. All 4YSZ ceramic specimens underwent air abrasion with 45 μm Al_2_O_3_ particles (Polidental, Cotia, São Paulo, Brazil) for 10 s under 2 bar pressure, employing oscillating movements at a distance of 1 cm between the surface and the blaster tip. Subsequently, the 4YSZ specimens were cleaned again in an ultrasonic bath and assigned to their respective experimental groups.

### Luting protocols and procedures

The protocols were implemented in accordance with the manufacturer's application recommendations and selected based on the experimental groups outlined in Table 1. The selection considered products available in the market, their compositions, and indications as follows:

1- Universal Primer: A universal primer containing MDP, disulfide, and silane (Monobond N, Ivoclar) was chosen. In this group, the primer was actively applied onto the 4YSZ surface for 15 s and volatilized for 45 s. Furthermore, only for the fatigue test, Primers A and B were mixed and actively applied to the etched dentin analogue for 30 s, followed by luting with Multilink N resin cement (Ivoclar AG).

2- Universal Adhesive: A universal adhesive containing MDP (Single Bond Universal adhesive; 3M ESPE Dental Products, St. Paul, MN, USA) was applied actively onto the 4YSZ surface for 20 s and volatilized for 5 s. Furthermore, only for the fatigue test, the adhesive was also applied on the etched dentin analogue. Luting was then carried out using RelyX Ultimate resin cement (3M ESPE).

3- Cement: A resin cement with MDP (Panavia F 2.0; Kuraray, Kurashiki, Okayama, Japan) was employed, by mixing equal amounts of pastes for 20 s before application onto the 4YSZ surface. Furthermore, only for the fatigue test, ED Primer II A and B were mixed and actively applied on the dentin analogue resin for 30 s, gently air-dried, and then luting was carried out with the resin cement.

4- Primer + Cement: This group utilized an VBATDT and MDP-containing primer (Alloy Primer; Kuraray) and resin cement (Panavia F 2.0, Kuraray). The primer was applied to the 4YSZ ceramics and left untouched for 45 s until the solvent was evaporated, followed by luting with the resin cement as described above.

For μSBS specimens, following surface treatments and primer applications, ten starch tubes (height= 1 mm, internal diameter= 1.2 mm) (Isabela, M. Dias Branco S.A. Indústria e Comércio de Alimentos, São Caetano do Sul, Brazil) were affixed with wax 7 on the luting surface. The resin cement from each experimental group was manipulated, applied inside the matrices using a resin spatula and endodontic spacer (#25, Dentsply Maillefer, Switzerland), and light cured for 20 s at close range (1200 mW/cm², Radii-Cal, SDI, Bayswater, Australia) in accordance with the manufacturer’s recommendations. Resulting in ten resin cement cylinders per ceramic slice.

For fatigue strength specimens, the resin cements were applied to the luting surface of the 4YSZ ceramic discs, which were then placed on the respective epoxy resin discs. Subsequently, a constant load of 2.5 N was applied, excess cement was removed, and the assembly was photoactivated for 20 s at close range (Radii-Cal, SDI) at each position (0°, 90°, 180°, 270°, and top).

### Aging

After luting, the specimens were immersed in distilled water at 37 ºC for 24 h. Following this, the starch tubes were removed, and the resin cement cylinders were examined. Any defects or bubbles observed at the interface resulted in the discarding of the respective cement cylinder. Post-evaluation, five specimens from the same ceramic slice underwent the microshear bond strength test, while the other five were subjected to an aging condition.

For the groups undergoing aging, storage occurred in distilled water at 37 ºC for 180 days, followed by exposure to 25,000 thermal cycles (5-55ºC, residence time 30 s, transfer time 5 s) using a thermal cycling machine (Nova Ética, Vargem Grande do Sul, SP, Brazil) ^13^.

Regarding the fatigue test, half of the specimens were stored in distilled water at 37ºC for 24 h prior to testing, while the other half underwent the aging protocols described above.

### Microshear bond streagth (μSBS) Test

The specimens were affixed to a universal testing machine (EMIC DL-2000, São José dos Pinhais, Brazil) and subjected to testing with a 100 N load cell at a speed of 1 mm/min using a stainless-steel wire (Ø= 0.2 mm) positioned on the adhesive interface. Bond strength (MPa) was calculated using the equation “S=L/A,” where “S” represents bond strength (MPa), “L” denotes the load at which the specimen failed (N), and “A” signifies the area of the adhesive interface (mm²).

### Cyclic fatigue test

The specimens underwent a cyclic fatigue test until failure using specialized equipment (Instron ElectroPuls E3000, Instron Corporation, Norwood, USA). In this test, cyclic loads were applied by a 40 mm diameter stainless steel hemispherical piston, with the sample immersed in water. To enhance contact and stress distribution, an adhesive tape (110 μm) was positioned between the piston and the luted assembly ^13^. A load frequency of 20 Hz was employed ^13^, with an initial 5,000 cycles at 200 N to adjust the piston/specimen contact. Subsequently, 15,000 cycles were performed at progressively increasing stress levels of 100 N, starting at 400 N, until crack detection by transillumination ^13^. The values for fatigue failure load (FFL) and the number of cycles to fatigue failure (CFF) were recorded for subsequent statistical analysis.

### Failure and fractographic analysis

Following the bond strength test, all specimens underwent analysis using a stereomicroscope (Stereo Discovery V20, Carl Zeiss, Göttingen, Germany). Failures were categorized as adhesive (involving more than 50% of the adhesive area) or cohesive (involving more than 50% of one substrate). Only adhesive failures were considered for statistical analysis. Representative images of each failure type were captured using Scanning Electron Microscopy (SEM - Vega3, Tescan, Czech Republic) at 100× magnification.

For the fatigue test, fragments from the failed specimens were highlighted to facilitate visualization of the failed region. Using a stereomicroscope (Discovery V20, Carl-Zeiss, Göttingen, Germany), the region of the crack and other fractographic characteristics were observed. Following this, a representative failed specimen was chosen for scanning electron microscopy analysis (Vega3, Tescan) at 250× and 1500× magnification to determine fracture origin, direction of crack propagation, and other pertinent fractographic features.

### Statistical analysis

The Kolmogorov-Smirnov test confirmed the normal distribution of the data. Bond strength data from adhesive failures underwent analysis through two-way ANOVA and Bonferroni post-hoc test (α= 0.05) using statistical software (IBM SPSS, Version 28.0, IBM Corp, Armonk, USA). Survival analysis, including Kaplan-Meier followed by Mantel-Cox post-hoc tests, was performed considering FFL and CFF (α= 0.05) using the same statistical software. Survival rates were calculated for all parameters at various testing steps. Qualitative analysis was employed for failure data.

## Results

In the Microshear Bond Strength test, two-way ANOVA and Bonferroni post-hoc tests (Table 1) revealed a statistically significant decrease in bond strength values for the Primer + Cement group (p<0.001), particularly after aging. The Cement group appeared stable after aging (p=0.303), while the Universal Adhesive and Universal Primer groups showed an increase in bond strength values (p<0.05).

**Table 1:** Microshear bond strength (μSBS) test results (Mean and standard deviations) in MPa and failures modes.

Group	Microshear bond strength		Failures (cohesive on cement/adhesive)	
	Baseline*	Aged*	Baseline	Aged
Universal Primer	22.2^B^ (5.5)	27.4^A^ (5.8)	0/50	0/50
Universal Adhesive	19.1^BC^ (6.0)	28.6^A^ (8.3)	1/49	0/50
Cement	15.3^CD^ (6.1)	12.2^D^ (6.7)	2/48	3/47
Primer + Cement	14.4^D^ (5.9)	5.4^E^ (4.7)	7/43	1/45

* Uppercase letters indicate statistical differences between evaluated conditions by two-way ANOVA and Bonferroni post-hoc tests (α= 0.05).

**Figure 2:**
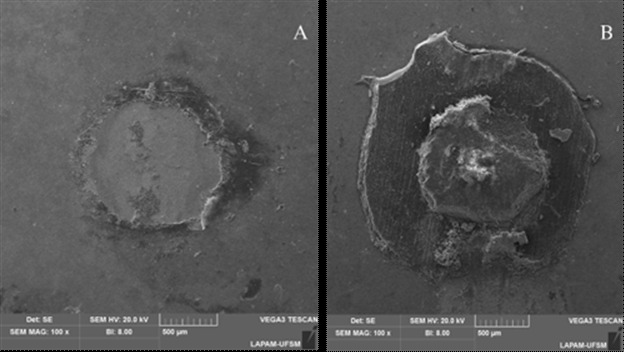
Representative SEM images (100 ×) of an adhesive failure (A) and a cohesive failure (B) from the μSBS test.

After aging, the Universal Adhesive and Universal Primer groups demonstrated statistically higher bond strength values compared to other groups (p<0.05); and the Cement group exhibited superior results compared to the Primer + Cement group (p<0.001) but inferior to the other groups (p<0.05). Pre-test failures were observed only in the Primer + Cement Aged group, resulting in the loss of a whole ceramic slice. Failure analysis (Figure 2) indicated a predominance of adhesive failures.

The percentage for the occurrence of cement cohesive failures relative to adhesive failures in the groups was as follows: 0%/100% in Universal Primer Baseline, 2%/98% in Universal Adhesive Baseline, 4%/96% in Cement Baseline, 14%/86% in Primer + Cement Baseline, 0%/100% in Universal Primer Aged, 0%/100% in Universal Adhesive Aged, 6%/94% in Cement Aged, and 2.2%/97.8% in Primer + Cement Aged (Table 1).

**Figure 3:**
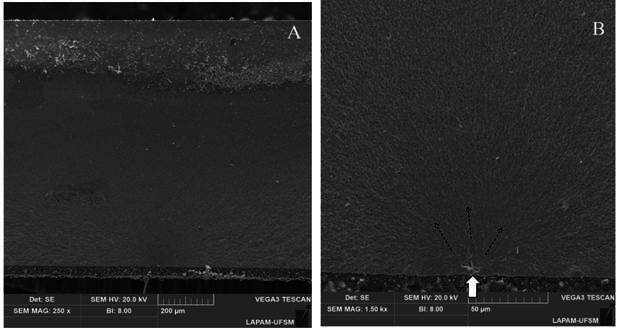
Representative SEM images at 250 × (A) and 1500 × (B). Failures originated at the luting surface from defects present at the ceramic surface (white arrow), and then propagated to the opposite side, as indicated by arrows (occlusal/top surface).

In terms of load-bearing capacity under fatigue, Kaplan-Meier and Mantel-Cox tests (Table 2) showed no statistically significant differences among the tested groups for both fatigue failure load and cycles for failure (p>0.05). The aging process did not lead to a decrease in fatigue strength data (p>0.05). Regarding failure patterns, radial cracks were observed in all tested specimens (Figure 3).

**Table 2:** Mean and 95% confidence interval for fatigue failure load (FFL) in Newtons and cycles for failure (CFF).

Group	FFL*		CFF*	
	Baseline	Aged	Baseline	Aged
Universal Primer	1263^Aa^ (1186 - 1339)	1367^Aa^ (1273 - 1460)	125,833^Aa^ (109,929 - 141,736)	148,333^Aa^ (129,694 - 166,972)
Universal Adhesive	1254^Aa^ (1159 - 1349)	1288^Aa^ (1108 - 1466)	119167^Aa^ (105496 - 132836)	132,221^Aa^ (96370 - 168073)
Cement	1267^Aa^ (1168 - 1366)	1315^Aa^ (1164 - 1465)	120,000^Aa^ (106,590 - 133,410)	136,667^Aa^ (108,927 - 164,406)
Primer + Cement	1246^Aa^ (1180 - 1311)	1329^Aa^ (1219 - 1440)	124,167^Aa^ (109,970 - 138,363)	140,734^Aa^ (118,536 - 162,932)

*Uppercase letters indicate statistical differences between evaluated conditions on columns, while lowercase letters indicate statistical differences between evaluated conditions on rows by Kaplan-Meier and Mantel-Cox post-hoc tests (α = 0.05).

## Discussion

This study investigated the adhesive and mechanical effects of using various luting systems containing MDP at the different components of the luting process, examining its additive impact on bonding to a 4YSZ ceramic. In the adhesive test, favorable behavior was observed in systems utilizing a universal primer or universal adhesive with MDP, combined with a non-MDP resin cement. However, the combination of different products with MDP did not show a favorable outcome. Thus, the first tested hypothesis was partially rejected. In contrast, the fatigue mechanical test revealed no differences among the used systems, indicating that the presence of MDP in a specific step or component, or its additive effect, is not a significant factor for this outcome. Consequently, the second tested hypothesis was rejected.

The current study observed that the group using an MDP primer in conjunction with an MDP-containing resin cement exhibited the poorest bond strength results to a 4YSZ. Similar findings were obtained by Moura et al., (2018) for a different zirconia ceramic, where luting systems with an MDP-containing primer or adhesive and MDP-free cements yielded better results than a combination of components with MDP ^14^. Additionally, existing literature suggests a potential plateau in MDP concentration ^15^, challenging prior assumptions ^7^. Whether by successive MDP primer applications ^15^, or by the combination of a primer and cement containing MDP ^16^, it seems that the extra monomer quantity is, at least, ineffective. Perhaps in these scenarios, the excess of MDP components will not have available zirconia particles to bond, showing no additional or beneficial effect ^15^. The primer used in the Primer + Cement group also contains VBATDT, another chemical intended to bond to zirconia and appears to have a synergic effect with MDP when they are in specific concentrations ^17^. However, it is not clear by the manufacturer the specific concentration of each component in the used formulation. Furthermore, the presence of MDP in the Primer + Cement group in the present study was negatively affected by the aging protocols more than non-MDP resin cements with prior MDP-containing universal primer or universal adhesive application, which is also in accordance with the literature ^14^. When testing experimental MDP containing adhesives, no linear correlation was found between MDP concentration and bond strength ^18^. Furthermore, as the MDP concentration in each system varies, there is still no consensus on whether there is a limit for the MDP concentration over the zirconia surface.

It is well established that the utilization of luting systems containing MDP is associated with improved adhesion to zirconia, as highlighted in the systematic review by Özcan and Bernasconi (2015) ^2^. Even though, it was previously demonstrated that the use of MDP-containing components still leads to a decrease in bond strength values over time when employing an experimental primer or luting components featuring this monomer ^19^. This demonstrates the possibility of degradation and a long-term reduction in performance.

When considering the oral environment, humidity is an exposing factor that always must be considered. The constant saliva presence facilitates the adhesive layer degradation, compromising its chemical bonds and quality ^8^. This degradation probably occurred due to the breaking of the bonds via hydrolysis or due to an insufficient MDP concentration to sustain strong bonded chains ^19^, since the increase in the concentration of this monomer can lead to an improvement in the interactions between the resinous material and ceramics ^7^. In addition, when it comes to in vitro studies, it has been pointed out that long times and aggressive aging protocols lead to more relevant data considering adhesion outcomes ^8^ ^,^ ^10^.

One notable result from the present study is the increase in bond strength after aging in the Universal Primer and Universal Adhesive groups and possible reasons for this can be interpreted. The Universal Adhesive has in its composition a specific copolymer (Vitrebond copolymer) that is a methacrylate-modified polyalkenoic acid and is known to have a moisture-stabilizing effect. This was possible to provide enough time for the resin cement to continue its polymerization process and have a better interlocking effect in the ceramic topography, yielding better bond strength over time ^20^. In the Universal Primer group, a similar reason explains the bond strength improvement after aging. As the resin cement for this group has a greater degree of conversion than in the other groups ^21^, the provided micromechanical interlocking was improved over the interaction with high temperatures, providing additional polymerization and an increase in elastic modulus ^22^. Furthermore, both resin cements without MDP also have a lower Young’s Modulus (Multilink N: 6.3 GPa and RelyX Ultimate: 7.7 GPa) than the one containing MDP (Panavia F 2.0: 12 GPa) ^23^ ^,^ ^24^, which make them easier to penetrate the ceramic’s irregularities and promote better micromechanical interlocking.

Despite the aesthetic advancements in zirconia ceramics, their notable feature remains high mechanical strength. When examining the fatigue data, it becomes evident that the different luting systems, with variations in the MDP component, did not induce significant alterations. This can be attributed to the high elastic modulus of 4YSZ (210 GPa) ^25^. Consequently, there were no statistical differences among the tested groups, as the elastic modulus of the used resin cements (Multilink N: 6.3 GPa; RelyX Ultimate 7.7 GPa; Panavia F 2.0: 10.2 GPa) ^23^ ^,^ ^24^ (28,29)exhibited small variations when compared to the ceramic. Previous tests with 4YSZ discs in setups similar to the present study, with comparable thickness, have indicated that this ceramic is more influenced by the support material ^4^ than by differences in resin cement properties ^26^. Additionally, while water aging can potentially alter the resin cement's elastic modulus by carrying away water-soluble monomers and affecting tension distribution over the restoration ^8^, in the current study, this was not sufficient to modify the fatigue behavior of the tested groups.

Some limiting factors should be considered in the present study, such as the use of luting systems from different manufacturers, leading to variations in material composition and concentrations. Additionally, the Primer + Cement group employed components from the same manufacturer, although not indicated for use together. This was done to address the research question posed in the study. Furthermore, zirconia ceramics from different generations ^1^ may exhibit varying responses to the proposed techniques and require further investigation. While the study employed water storage and thermocycling aging methods, the oral environment is dynamic and aggressive. Models incorporating pH variations could yield different results. Despite utilizing a simplified disc setup for the mechanical test, this research stands as the first to evaluate adhesive outcomes with a fatigue strength test, assessing different luting strategies containing MDP.

## Conclusion

Luting systems with MDP-containing primers or adhesives paired with non-MDP resin cements demonstrated enhanced bond strength for 4YSZ in the long term. Despite that, all luting systems exhibited comparable fatigue behavior for 4YSZ bonded to an epoxy resin.
